# Tranexamic acid in shoulder surgery: a narrative review

**DOI:** 10.1016/j.xrrt.2026.100830

**Published:** 2026-07-22

**Authors:** Henrik A. Hahamyan, Peter S. Chang, Lance E. LeClere

**Affiliations:** a Quillen College of Medicine, East Tennessee State University, Johnson City, TN, USA; b Department of Orthopaedic Surgery, Vanderbilt University Medical Center, Nashville, TN, USA

**Keywords:** Tranexamic acid, Shoulder surgery, Shoulder arthroscopy, Shoulder arthroplasty, Perioperative, Orthopedic surgery, Antifibrinolytic

## Abstract

Tranexamic acid (TXA) has become a popular perioperative adjunct in orthopedic surgery for its ability to reduce total blood loss, transfusion rates, and associated complications. Although most data are within total hip and knee arthroplasty along with other open procedures, TXA in shoulder surgery remains less certain. Shoulder surgery encompasses a broad spectrum of techniques from minimally invasive arthroscopy to a variety of open approaches that each have distinct soft tissue planes, operative exposures, and opportunities for bleeding. Each procedure carries its own bleeding profiles, perioperative considerations, and outcomes of interest, and data for TXA indications vary accordingly. In open shoulder surgery, TXA is used to minimize intraoperative and post-operative blood loss, while in arthroscopic surgery, it helps limit bleeding for better visualization. This narrative review seeks to evaluate the current evidence of TXA use in shoulder surgery and provide a practical resource for surgeons and perioperative teams involved in management.

Introduced in the 1960s, the use of tranexamic acid (TXA) has gained popularity over the past 20 years for its ability to decrease bleeding and associated complications.^59^ Because substantial intraoperative and post-operative blood loss may occur in a variety of elective orthopedic surgeries, TXA has become a favorable adjunct among surgeons and has been increasingly used in joint arthroplasty and spine surgery. From 2016 to 2020, TXA was administered in approximately 78% of 1.1 million total hip and knee arthroplasty cases.^35^ While most evidence is in hip and knee arthroplasty, its use has expanded across nearly all orthopedic subspecialties, including trauma, sports medicine, adult reconstruction, spine, pediatrics, foot and ankle, and oncology.^1^^,^^20^^,^^23^^,^^26^^,^^27^^,^^29^^,^^37^^,^^62^

As a synthetic, reversible antifibrinolytic agent originally designed in Japan for postpartum hemorrhage, TXA competitively inhibits the lysine receptor found on plasminogen as part of the fibrinolytic system.^54^^,^^67^ The binding of this receptor prevents the activated form of plasminogen from binding and degrading the fibrin clot, leading to the maintenance of formed blood clots. As a fibrin clot stabilizer, this mechanism may prevent bleeding but naturally raises concern for venous thromboembolism (VTE) complications.

Despite its widespread use in many high-volume orthopedic procedures, notable gaps remain regarding the settings in which TXA is most effective, the most relevant primary outcomes, and the optimal route and dosing strategy. Shoulder surgery, in particular, represents an area of ongoing uncertainty given its wide range of surgical approaches (open, arthroscopic, and combined), each with different bleeding profiles and perioperative considerations. Accordingly, the goal of this narrative review is to evaluate the current evidence of TXA use in orthopedic shoulder surgery and provide a practical resource for surgeons and perioperative teams involved in management.

## Background

To date, TXA has only 2 U.S. Food and Drug Administration–approved indications with 2 routes of administration. The intravenous (IV) formulation, CYKLOKAPRON (Pfizer, New York, NY, USA), was approved in 1986 for use in patients with hemophilia to prevent hemorrhage during and following tooth extraction dosed at 10 mg/kg IV before and 3-4 times daily after surgery.^16^ The oral formulation, LYSTEDA (Ferring Pharmaceuticals, Saint-Prex, Switzerland), was approved in 2009 for the treatment of cyclic heavy menstrual bleeding in females of reproductive potential, administered as 1300 mg 3 times daily for up to 5 days during monthly menstruation.^48^

Beyond these 2 indications, TXA is used off-label in a variety of conditions, including acute upper gastrointestinal bleeding, postpartum hemorrhage, cesarean section, trauma resuscitation, prostate surgery, and cardiac surgery.^7^^,^^12^^,^^14^^,^^31^^,^^36^^,^^45^^,^^51^ In orthopedics, TXA was first used in 1995, investigating reductions in blood loss in total knee arthroplasty (TKA).^8^ Since then, over 120 randomized control trials (RCTs) have been published on the use of TXA in orthopedic surgery.

## Non-shoulder orthopedic surgery

The strongest evidence for TXA in orthopedic surgery likely remains in TKA and total hip arthroplasty (THA). In 2018, an evidence-based clinical practice guideline jointly endorsed by the American Association of Hip and Knee Surgeons, the American Society of Regional Anesthesia and Pain Medicine, the American Academy of Orthopaedic Surgeons, The Hip Society, and The Knee Society provided data-driven recommendations regarding the use of TXA in TKA and THA.^25^ The guidelines specified that all formulations (IV, topical, oral) are effective and not superior to each other, there is no specific TXA dose response, multiple vs. single doses of TXA do not significantly alter outcomes, and administration before incision may be more effective than after incision.^24^ Furthermore, the 2023 American Academy of Orthopaedic Surgeons guidelines on the management of hip osteoarthritis provide the highest level of recommendation for the use of TXA in patients undergoing THA to reduce blood loss and transfusions.^4^

Anterior cruciate ligament reconstruction (ACLR) has also been heavily investigated. In a 2021 systematic review, IV TXA in ACLR was found to have reduced post-operative drain output, improved clinical hemarthrosis grades, visual analog scale (VAS) pain scores, range of motion (ROM), and Lysholm functional scores up to 6 weeks post-operatively with no greater risk of VTE or other medical complications.^44^

TXA has also been explored in numerous fracture patterns and fixation techniques. RCTs with TXA in calcaneus, intertrochanteric femur, femoral neck, and tibia fractures identified reductions in post-operative blood loss and/or transfusion rates.^19^^,^^52^^,^^71^^,^^72^ Systematic reviews evaluating TXA in pelvic/acetabular fracture surgery, hemiarthroplasty for femoral neck fracture, and intertrochanteric femur fracture with intramedullary fixation report similar findings.^43^^,^^63^^,^^68^^,^ The apparent universal benefit of TXA in common orthopedic trauma procedures likely reflects an ideal combination of the vascular nature of injuries and the antifibrinolytic mechanism.

Use of TXA in elbow, hip, and knee arthroscopy has also been investigated, though evidence remains limited. In arthroscopic meniscectomy, an RCT with IV TXA reported improvement in the Tegner score 3 days post-operatively but found no differences in pain, ROM, or swelling.^53^ A cohort study of intra-articular (IA) TXA in arthroscopic knee arthrolysis found decreased VAS pain scores, improved ROM and Lysholm scores, and lower inflammatory markers in the TXA group.^47^ Beyond the knee, an RCT of IV TXA in hip arthroscopy for femoroacetabular impingement syndrome found no improvement in visual field clarity, while another cohort study in general hip arthroscopy showed statistically significant, but likely not clinically meaningful reduction in arthroscopic fluid utilization and operative time in the TXA group.^46^^,^^61^ In a cohort study and RCT for elbow arthroscopy, IV and IA TXA reduced post-operative blood loss but no changes in ROM or pain.^21^^,^^78^ Overall, IV TXA shows mixed benefit across non-shoulder arthroscopy and may vary by joint type. IA TXA may offer some promise, though several preclinical studies in animal models have reported cytotoxicity, potentially limiting its clinical applicability.^11^^,^^61^

Given that patients undergoing orthopedic surgery already have an elevated baseline risk of VTE, the addition of an antifibrinolytic has raised concerns for complications.^55^ However, numerous studies across general and orthopedic populations have continuously demonstrated no increased risk of VTE.^41^^,^^50^^,^^55^^,^^60^ Furthermore, the aforementioned jointly endorsed evidence-based clinical practice guideline describes no increased risk of VTE in patients without a history of VTE in primary total joint arthroplasty.^25^ Even among patients who have a history of prior VTE, myocardial infarction, cerebrovascular accident, and/or vascular stent placement, although RCTs are limited, the existing high-quality literature does not suggest increased risk of thromboembolic events in these patients.^25^ In these cases, the clinical practice guideline recommends individualized decision-making with a multidisciplinary approach.^25^

## Open shoulder surgery

### Shoulder arthroplasty

The use of TXA in total and reverse shoulder arthroplasty (TSA, rTSA) has risen substantially over the past 2 decades, increasing from use in 0.4% of cases in 2010 and 41.2% in 2016 to 60.3% in 2020.^5^^,^^50^ In a national cohort investigation of TXA in primary TSA and rTSA between 2016 and 2020, the risk of transfusion was lower for those who received TXA with no significant differences in bleeding, medical, surgical, or infectious complications in addition to rates of revision or readmission.^50^ This cohort data is supported further by a meta-analysis for TXA in primary TSA and rTSA, which identified a significant decrease in need for transfusion, blood loss in drainage, and total blood loss among those receiving TXA compared to not.^34^

Beyond blood-related parameters, a cohort investigation with shoulder arthroplasty (anatomic, hemi, reverse) identified a significant increase in post-operative shoulder elevation at 6 months among those who received an unknown route of TXA after controlling for covariates.^56^ Despite this benefit, there were no changes in external rotation, pain, need for physical therapy, or blood-related outcomes. Improvement in ROM has been described with IV and IA TXA in TKA and attributed to decreased post-operative hemarthrosis and swelling, which may explain the observed findings in TSA.^18^

Regarding the route of administration, the aforementioned national cohort investigation only included IV TXA and demonstrated effectiveness with reduced transfusion rates in TSA.^50^ The efficacy of IA TXA appears mixed with conflicting results among 2 RCTs.^28^^,^^73^ When compared directly to each other, a study comparing IA to IV TXA in TSA found no differences in transfusion rate, total blood loss, and total drain output.^76^ IA TXA administration varies across studies, which may include subacromial placement before closure, infusion through post-operative drains, and use of different dilution volumes. These inconsistencies make comparison with the more standardized pre-incision IV dosing difficult, and differences in timing during the procedure may further influence outcomes independent of route. Furthermore, as previously discussed, preclinical knee research indicates that TXA may exert a cytotoxic effect on articular cartilage at higher doses, though it remains unclear whether this extends to the shoulder.^11^^,^^62^

An RCT comparing oral and IV TXA in TSA and rTSA found no significant differences in hemoglobin change, drain output, length of stay, or transfusion rate.^26^ These findings align with the aforementioned 2018 evidence-based clinical practice guideline for THA and TKA.^25^ Moreover, cost-benefit analyses in THA and TKA have shown significant savings associated with the oral formulation.^22^^,^^69^ Although comparable economic analyses in oral TXA are lacking, given the similar dosing, absence of a clearly superior formulation, and potential cytotoxicity and lack of clear efficacy with IA TXA, the oral route may offer practical and economic advantages in shoulder arthroplasty.

### Latarjet

The Latarjet procedure for anterior shoulder instability has been evaluated in one RCT with 1g IV TXA vs. placebo. The study found that TXA significantly reduced post-operative blood loss, painful post-operative swelling, opioid consumption, and post-operative VAS pain scores compared with placebo.^38^ Beyond this RCT, no other studies have investigated TXA for Latarjet or other anterior shoulder instability surgeries.

### Fracture repair

TXA has also been investigated with proximal humerus fracture (PHF) repair, either with open reduction internal fixation (ORIF) or rTSA. Two RCTs investigated PHF repair with ORIF and rTSA, identifying significant reductions in post-operative blood loss with IV TXA compared to placebo.^15^^,^^74^ Furthermore, one cohort study with IA TXA in rTSA for PHF repair reported comparable reductions in blood loss, along with decreased transfusion rates and shorter hospital length of stay.^9^ In contrast, another cohort study in a similar setting found no significant differences with IV TXA.^58^ Consistent with findings from other fracture sites, perioperative IV TXA appears to reduce blood loss in PHF repair with ORIF; however, there remains mixed cohort data with rTSA, and further RCTs and a meta-analysis may help clarify these effects.

Beyond primary TSA/rTSA, Latarjet, and PHF repair, no other open shoulder surgeries have been investigated with TXA (Table I). Specifically, evidence is lacking for its use in other open shoulder instability surgeries, tendon transfers, shoulder arthroplasty revisions, open rotator cuff repair, open acromioclavicular reconstruction, clavicular ORIF, and pectoralis major repair.

**Table I:** Summary of studies on open shoulder surgery with TXA.

Procedure	TXA route/dose	Relevant outcomes compared to control	Conclusion
Shoulder arthroplasty	Mixed	In a meta-analysis, TXA decreased transfusion rate and total blood loss. In a cohort study, TXA increased shoulder elevation at 6 mo post-operatively, but no other recorded changes^50^^,^^56^	In TSA/rTSA, TXA is effective in reducing transfusion rate and post-operative blood loss. Results with IA TXA are mixed. Oral TXA may be equally effective to IV TXA and also may have a reduced cost to patients.
	IV	In a national cohort study, TXA lowered transfusion rate and did not increase bleeding, medical, surgical, or infectious complications^50^	
	IA, 1-2g	One cohort study demonstrated 1g IA TXA did not reduce blood loss; one RCT with 2g IA TXA reduced post-operative blood loss; one RCT showed equivalent reductions in post-operative blood loss when directly comparing 1g IV and 2g IA TXA^28^^,^^73^^,^^76^	
	Oral, 2g	In an RCT comparing 2g oral and 1.5 g IV TXA, reductions in post-operative blood loss were equivalent^26^	
Latarjet	IV, 1g	In one RCT, TXA reduced post-operative blood loss, swelling, opioid consumption, and pain^38^	In Latarjet, IV TXA is effective in improving blood-related outcomes and pain
PHF repair			
ORIF	IV, 1g	In two RCTs, TXA reduced intraoperative and post-operative blood loss with no change in complication or transfusion rate^15^^,^^74^	In ORIF for PHF, IV TXA is effective in reducing blood loss but not transfusion rate
rTSA	IV, 1g	In one RCT, TXA reduced total blood loss with no change in complication rate, while one cohort study did not show a change in post-operative blood loss^15^^,^^58^	In rTSA for PHF, IA TXA is effective in reducing blood-related parameters, while results are mixed for IV TXA
	IA, 1g	In one RCT, TXA reduced total blood loss, transfusion rate, and hospital length of stay^9^	

*IA*, intra-articular; *IV*, intravenous; *ORIF*, open reduction internal fixation; *PHF*, proximal humerus fracture; *RCT*, randomized control trial; *rTSA*, reverse total shoulder arthroplasty; *TXA*, tranexamic acid; *TSA*, total shoulder arthroplasty.

## Arthroscopic shoulder surgery

### Arthroscopic rotator cuff repair

Given minimal blood loss in arthroscopy compared to the other discussed approaches, the focus of TXA research shifts away from blood loss parameters (Table II).^30^ At least 10 systematic reviews have been published on TXA in arthroscopic rotator cuff repair (ARCR) between 2022 and 2025.^2^^,^^3^^,^^17^^,^^33^^,^^39^^,^^42^^,^^49^^,^^57^^,^^64^^,^^77^ Although most systematic reviews agree there were significant improvements in intraoperative visual clarity and operating time, the most recent systematic review emphasized that although the benefits identified may be statistically significant, they are likely not clinically significant.^17^ Fortunately, one of the systematic reviews noted that IV or IA TXA did not significantly increase the risk of complications or thromboembolic events.^64^ Following the most recent systematic review, a new RCT reported that IV TXA after ARCR reduced post-operative opioid consumption by 18 morphine milligram equivalents without affecting subjective pain scores, mirroring the mixed pain outcomes previously noted in the reviews.^13^

**Table II:** Summary of studies on arthroscopic shoulder surgery with TXA.

Procedure	TXA route/dose	Relevant outcomes compared to control	Conclusion
ARCR	Mixed	Across 10 SRs, a majority describe improvements in visual clarity during surgery, although potentially not clinically significant, but have mixed results for post-operative pain and operative time.^2^^,^^3^^,^^17^^,^^33^^,^^39^^,^^42^^,^^49^^,^^57^^,^^64^^,^^77^ One SR described no increase in VTE or other complications with TXA, while another specified that IV TXA was more effective at increasing visual clarity and reducing operative time than IA TXA.^9^^,^^64^	In ARCR, TXA may improve intraoperative visual clarity, post-operative pain, and post-operative opioid consumption, but more research is needed to identify clinical significance. IV TXA does not increase risk of complications, and IV may be more effective than IA.
	IV, 1g	In one RCT, TXA reduced post-operative opioid consumption but no changes in pain.^13^	
	IA, 250 mg-2g	Across 4 RCTs with IA TXA, results on pain, visual clarity, and blood loss were mixed, with potential for functional benefit.^13^^,^^40^^,^^42^^,^^79^ One RCT compared IV with IA TXA and found improvement in visual clarity vs. control but no differences between routes.^70^	
Capsule release	IA, 1g	In a cohort study for adhesive capsulitis, IA TXA plus cocktail∗ led to reduced drainage, pain, and improved functional scores compared to cocktail∗ alone.^6^	In arthroscopic capsule release, IA TXA with a cocktail∗ may improve a variety of post-operative outcomes, but no RCTs have been performed with TXA alone in this procedure
Other	IV, 1g	In a pooled group of multiple arthroscopic shoulder surgeries in one RCT, IV TXA did not improve visual clarity or post-operative pain, while epinephrine alone and combined with TXA improved visual clarity alone.^65^	In a pooled group of arthroscopic shoulder surgeries, IV TXA alone may not improve intraoperative visual clarity and post-operative pain.

*ARCR*, arthroscopic rotator cuff repair; *IA*, intra-articular; *IV*, intravenous; *RCT*, randomized control trial; *SR*, systematic review; *TXA*, tranexamic acid; *VTE*, venous thromboembolism.

∗ Cocktail included betamethasone, ropivacaine, flurbiprofen, and morphine.

Regardless, as also described in an editorial, substantial methodological variation across studies limits the strength of conclusions supporting TXA for visual clarity or reduced operative time.^40^ Notable differences in the included RCTs involve the number and expertise of surgeons involved, visual clarity and pain measurement method, and TXA dose/route and utilized adjuncts. However, given the low risk with possible benefit with potential pain placebo effects, IV TXA may be effective in ARCR for visual clarity, operative time, and pain, but future large-scale, standardized RCTs with specific patient subgroups should be performed for further clarity. It also remains unclear whether TXA influences non–bleeding-related outcomes, and further research may delineate its impact on healing, functional recovery, and other associated factors.

Regarding route, both IV and IA approaches have been evaluated in ARCR, while no studies have examined oral TXA. One systematic review noted that IV TXA improved visual clarity and operating time, whereas IA TXA did not.^77^ Among 4 RCTs investigating IA TXA, results were mixed: 2 demonstrated benefits with reduced pain, improved visual clarity, or decreased blood loss, while 2 found no significant post-operative changes, specifically no reductions in swelling or pain.^10^^,^^32^^,^^66^^,^^79^ Notably, 1 study reporting no differences in pain still identified significantly improved functional outcomes at 6 months compared to placebo.^32^ One RCT directly comparing IV TXA with TXA in irrigation fluid found improved visual clarity vs. control but no differences between routes.^70^ Although some non-shoulder arthroscopy models suggest IA TXA cytotoxicity, an ARCR preclinical model demonstrated adhesion reduction without adverse effects on collagen or tendon–bone healing.^75^ Overall, IA TXA may offer benefits in visualization and pain, but these effects appear less consistent than with IV administration and require further investigation.

### Capsule release

One study evaluated TXA in arthroscopic capsular release for adhesive capsulitis, using it as an adjunct to an IA “cocktail” of betamethasone, ropivacaine, flurbiprofen, and morphine.^6^ TXA with the cocktail was delivered via drainage catheter immediately after surgery and was associated with reduced post-operative drainage, pain, and improved 1-week functional scores compared to the cocktail alone. However, the absence of a true placebo group limited conclusions regarding the effect of TXA alone. While TXA may serve as an adjunct, dedicated studies isolating TXA in capsular release are needed.

### Other

Beyond the aforementioned arthroscopic procedures, an RCT investigated IV TXA for visual clarity in a pooled group of arthroscopic shoulder surgeries ranging from ARCR to distal clavicle excision.^65^ Pooled analysis showed no improvement in visual clarity or pain with 1g IV TXA compared to placebo, while 0.33 mL of 1:1000 per liter of epinephrine alone improved visual clarity. Given differences between arthroscopic shoulder surgeries, further research is needed to identify the role of TXA. Specifically, research is needed in arthroscopic labral repair and capsulorrhaphy, subacromial decompression, biceps tenodesis, and/or débridement procedures.

## Conclusion

Although a relatively newer intervention, TXA carries numerous off-label applications across orthopedics. Evidence is strongest in total hip and knee arthroplasty, followed by numerous fracture patterns and ACLR. In open shoulder surgery, IV TXA demonstrates robust reductions in blood loss and transfusion rates in primary TSA/rTSA, with questionable functional benefit; oral formulations offer equivalent efficacy at lower cost. Similar benefits have been reported in Latarjet procedures and PHF repair with ORIF or rTSA, though data for other open shoulder operations remain minimal.

In arthroscopic shoulder surgery, most studies focus on ARCR, where TXA statistically improves visual clarity and pain, and reduces operative time, though the clinical significance of these effects remains questionable. IV TXA may be beneficial in ARCR but requires more rigorous, subgroup-specific trials, and additional investigation is needed across other specific, arthroscopic procedures. Overall, TXA appears advantageous in TSA/rTSA, Latarjet, and PHF repair, yet high-quality clinical research is essential to delineate its role across the full spectrum of open and arthroscopic shoulder surgery.

## Disclaimers:

Funding: No funding was disclosed by the authors.

Conflicts of interest: Lance E. LeClere is a consultant for Arthrex and Vericel; serves on the board of directors for AOSSM; serves on the executive committee of the Team Physician Consensus Conference; is the chair of the NHL project review committee; and serves on the editorial board for AJSM, VJSM, and Arthroscopy. Affiliated with them, fellowship grant funding is supported by Enovis and departmental general research support is received from Ossio. Any additional authors, their immediate families, and any research foundations with which they are affiliated have not received any financial payments or other benefits from any commercial entity related to the subject of this article.

## References

[1] Alaia M.J., Gipsman A.M. (2021). Editorial commentary: the benefits of tranexamic acid May outweigh risks in arthroscopy and sports medicine. Arthrosc J Arthrosc Relat Surg.

[2] Alyousef M.Y., Alaqaili S.I., Alzayer M.A., Alsultan A.S., Absultan A.J., Alzahrani M.M. (2024). The efficacy of preoperative tranexamic acid administration among patients undergoing arthroscopic rotator cuff repair: a systematic review and meta-analysis of randomized controlled trials. Shoulder Elb.

[3] Alzobi O.Z., Derbas J., Toubasi A., Hantouly A., Abdullah A., Zikria B. (2024). Tranexamic acid use in arthroscopic rotator cuff repair: a systematic review and meta-analysis of randomized controlled trials. JSES Int.

[4] (2023). American Academy of orthopaedic surgeons management of osteoarthritis of the Hip Evidence-Based Clinical Practice Guideline. American Academy of Orthopaedic Surgeons. https://www.orthoguidelines.org/guideline-detail?id=1815&tab=all_guidelines.

[5] Anthony S.G., Patterson D.C., Cagle P.J., Poeran J., Zubizarreta N., Mazumdar M. (2019). Utilization and real-world effectiveness of tranexamic use in shoulder arthroplasty: a population-based study. J Am Acad Orthop Surg.

[6] Bai X., Bai F., Wang Z., Zhang Y., Liu F., Wang Q. (2023). Clinical efficacy of intra-articular infusion of cocktail combined with tranexamic acid in the treatment of middle-age and older patients with frozen shoulder following arthroscopic capsular release. Front Surg.

[7] Bennett C., Klingenberg S.L., Langholz E., Gluud L.L. (2014). Tranexamic acid for upper gastrointestinal bleeding. Cochrane Upper GI and Pancreatic Diseases Group. Cochrane Database Syst Rev.

[8] Benoni G., Carlsson A., Petersson C., Fredin H. (1995). Does tranexamic acid reduce blood loss in knee arthroplasty?. Am J Knee Surg.

[9] Beyth S., Fraind-Maya G., Safran O. (2023). Tranexamic acid treatment reduces blood loss after elective and semi-urgent reverse total shoulder arthroplasty. Geriatr Orthop Surg Rehabil.

[10] Bildik C., Pehlivanoglu T. (2023). Arthroscopic rotator cuff repair performed with intra-articular tranexamic acid: could it provide improved visual clarity and less postoperative pain? A prospective, double-blind, randomized study of 63 patients. J Shoulder Elbow Surg.

[11] Bolam S.M., O’Regan-Brown A., Paul Monk A., Musson D.S., Cornish J., Munro J.T. (2021). Toxicity of tranexamic acid (TXA) to intra-articular tissue in orthopaedic surgery: a scoping review. Knee Surg Sports Traumatol Arthrosc.

[12] Bouras M., Bourdiol A., Rooze P., Hourmant Y., Caillard A., Roquilly A. (2024). Tranexamic acid: a narrative review of its current role in perioperative medicine and acute medical bleeding. Front Med.

[13] Burns K.A., Robbins L.M., Humphrey L.A., LeMarr A.R., Morton D.J., Wilson M.L. (2025). Use of tranexamic acid reduces opioid consumption after arthroscopic rotator cuff repair. J Shoulder Elbow Surg.

[14] CRASH-2 trial collaborators (2010). Effects of tranexamic acid on death, vascular occlusive events, and blood transfusion in trauma patients with significant haemorrhage (CRASH-2): a randomised, placebo-controlled trial. Lancet.

[15] Cuff D.J., Simon P., Gorman R.A. (2020). Randomized prospective evaluation of the use of tranexamic acid and effects on blood loss for proximal humeral fracture surgery. J Shoulder Elbow Surg.

[16] (2021). CYKLOKAPRON® (tranexamic acid) injection, for intravenous use. U.S. Food and Drug Administration. https://www.accessdata.fda.gov/drugsatfda_docs/label/2021/019281s047lbl.pdf.

[17] Dagher D., Kashir I., Mahboob O., Al-Turki N., Khan M. (2025). Tranexamic acid has a limited role in improving visual clarity and pain in arthroscopic shoulder surgery: a systematic review and meta-analysis. Arthrosc J Arthrosc Relat Surg.

[18] Dorweiler M., Boin M., Froehle A., Lawless M., May J. (2019). Improved early postoperative range of motion in total knee arthroplasty using tranexamic acid: a retrospective analysis. J Knee Surg.

[19] Drakos A., Raoulis V., Karatzios K., Doxariotis N., Kontogeorgakos V., Malizos K. (2016). Efficacy of local administration of tranexamic acid for blood salvage in patients undergoing intertrochanteric fracture surgery. J Orthop Trauma.

[20] Edelstein A., McDonald J., Lachance A.D., Giro M.E., Lee W. (2023). The efficacy and safety of tranexamic acid utilization in total ankle arthroplasty: a systematic review and meta-analysis. Arch Orthop Trauma Surg.

[21] Ek E.T., Wang K.K., Bohan C.M., Goulding N.J., Jamieson R.P. (2022). Role of tranexamic acid in arthroscopic osteocapsular release of the elbow for degenerative arthritis. Orthop J Sports Med.

[22] Fillingham Y.A., Kayupov E., Plummer D.R., Moric M., Gerlinger T.L., Della Valle C.J. (2016). The James A. Rand young investigator’s award: a randomized controlled trial of oral and intravenous tranexamic acid in total knee arthroplasty: the same efficacy at lower cost?. J Arthroplasty.

[23] Fillingham Y.A., Ramkumar D.B., Jevsevar D.S., Yates A.J., Shores P., Mullen K. (2018). The efficacy of tranexamic acid in total hip arthroplasty: a network meta-analysis. J Arthroplasty.

[24] Fillingham Y.A., Ramkumar D.B., Jevsevar D.S., Yates A.J., Shores P., Mullen K. (2018). The safety of tranexamic acid in total joint arthroplasty: a direct meta-analysis. J Arthroplasty.

[25] Fillingham Y.A., Ramkumar D.B., Jevsevar D.S., Yates A.J., Bini S.A., Clarke H.D. (2018). Tranexamic acid use in total joint Arthroplasty: the Clinical Practice Guidelines endorsed by the American Association of hip and knee surgeons, American Society of regional anesthesia and pain medicine, American Academy of orthopaedic surgeons, hip Society, and knee society. J Arthroplasty.

[26] Gao R., Hirner M., Van Niekerk M., Ledesma E., Gibson A., Campbell A. (2022). Oral and intravenous tranexamic acid are equivalent at reducing blood loss following shoulder arthroplasty—A multicenter, double-blinded, randomized, placebo-controlled trial. Semin Arthroplasty.

[27] Gausden E.B., Qudsi R., Boone M.D., O’Gara B., Ruzbarsky J.J., Lorich D.G. (2017). Tranexamic acid in orthopaedic trauma surgery: a meta-analysis. J Orthop Trauma.

[28] Gillespie R., Shishani Y., Joseph S., Streit J.J., Gobezie R. (2015). Neer Award 2015: a randomized, prospective evaluation on the effectiveness of tranexamic acid in reducing blood loss after total shoulder arthroplasty. J Shoulder Elbow Surg.

[29] Goobie S.M., Zurakowski D., Glotzbecker M.P., McCann M.E., Hedequist D., Brustowicz R.M. (2018). Tranexamic acid is efficacious at decreasing the rate of blood loss in adolescent scoliosis surgery: a randomized placebo-controlled trial. J Bone Joint Surg Am.

[30] Griffiths D., Jagiello J., Dheerendra S., McNamara C., Ahrens P. (2013). Blood loss in arthroscopic shoulder surgery: is it clinically important?. Shoulder Elb.

[31] Gungorduk K., Yıldırım G., Asıcıoğlu O., Gungorduk O., Sudolmus S., Ark C. (2011). Efficacy of intravenous tranexamic acid in reducing blood loss after elective cesarean section: a prospective, randomized, Double-Blind, placebo-controlled study. Am J Perinatol.

[32] Guo J., Que M., Guo J., Liu Z., Che Y.J. (2025). A therapeutic assessment of tranexamic acid on functional recovery after rotator cuff repair surgery: a study of early and mid-term follow-up. J Orthop.

[33] Han C., Liu M., Lian X., Sun T., Yan S., Bai X. (2023). Tranexamic acid use in arthroscopic rotator cuff repair is an effective and safe adjunct to improve visualization: a systematic review and meta-analysis. J Shoulder Elbow Surg.

[34] He J., Wang X., Yuan G., Zhang L. (2017). The efficacy of tranexamic acid in reducing blood loss in total shoulder arthroplasty: a meta-analysis. Medicine (Baltimore).

[35] Heckmann N.D., Haque T.F., Piple A.S., Mayfield C.K., Bouz G.J., Mayer L.W. (2023). Tranexamic acid and prothrombotic complications following total hip and total knee arthroplasty: a population-wide safety analysis accounting for surgeon selection bias. J Arthroplasty.

[36] Hernando Mina S., Garcia-Perdomo H.A. (2018). Effectiveness of tranexamic acid for decreasing bleeding in prostate surgery: a systematic review and meta-analysis. Cent Eur J Urol.

[37] Hess M.C., Andrews N.A., Crowley B., Singh N.P., Howie C., McGwin G. (2023). Intravenous tranexamic acid decreases intraoperative transfusion requirements and does not increase incidence of symptomatic venous thromboembolic events in musculoskeletal sarcoma surgery. Surg Oncol.

[38] Hurley E.T., Lim Fat D., Pauzenberger L., Mullett H. (2020). Tranexamic acid for the Latarjet procedure: a randomized controlled trial. J Shoulder Elbow Surg.

[39] Hurley E.T., Rodriguez K., Karavan M.P., Levin J.M., Helmkamp J., Anakwenze O. (2024). Tranexamic acid for Rotator Cuff Repair: a systematic review and meta-analysis of randomized controlled trials. Am J Sports Med.

[40] Hurley E.T. (2025). Editorial commentary: tranexamic acid for shoulder Arthroscopy shows low cost, low risk, small benefits, and meaningful potential to reduce postoperative hemarthrosis and operative time. Arthrosc J Arthrosc Relat Surg.

[41] Ivasyk I., Chatterjee A., Jordan C., Geiselmann M.T., Chang P.S., Kamel H. (2022). Evaluation of the safety of tranexamic acid use in pediatric patients undergoing spinal fusion surgery: a retrospective comparative cohort study. BMC Musculoskelet Disord.

[42] Jain N., McKeeman J., Schultz K., Chan W., Aaron D., Busconi B. (2025). Tranexamic acid use in rotator cuff repair: a systematic review of perioperative outcomes. J Orthop.

[43] Jiang J., Xing F., Zhe M., Luo R., Xu J., Duan X. (2022). Efficacy and safety of tranexamic acid for patients with intertrochanteric fractures treated with intramedullary fixation: a systematic review and meta-analysis of current evidence in randomized controlled trials. Front Pharmacol.

[44] Johns W.L., Walley K.C., Hammoud S., Gonzalez T.A., Ciccotti M.G., Patel N.K. (2021). Tranexamic acid in anterior cruciate ligament reconstruction: a systematic review and meta-analysis. Am J Sports Med.

[45] Ker K., Sentilhes L., Shakur-Still H., Madar H., Deneux-Tharaux C., Saade G. (2024). Tranexamic acid for postpartum bleeding: a systematic review and individual patient data meta-analysis of randomised controlled trials. Lancet.

[46] Kunze K.N., Madjarova S., Olsen R., Smolarsky R., Menta S., Baldwin R. (2025). Intravenous tranexamic acid does not improve visual field clarity during hip arthroscopy: a Double-Blind, randomized, placebo-controlled trial. Arthrosc J Arthrosc Relat Surg.

[47] Li J., You M., Yao L., Fu W., Li Q., Chen G. (2023). Topical administration of tranexamic acid reduces postoperative blood loss and inflammatory response in knee arthroscopic arthrolysis: a retrospective comparative study. BMC Musculoskelet Disord.

[48] (2020). LYSTEDA® (tranexamic acid) tablets, for oral use. U.S. Food and Drug Administration. https://www.accessdata.fda.gov/drugsatfda_docs/label/2020/022430s009lbl.pdf.

[49] Malik S.S., Tahir M., Jordan R.W., Kwapisz A., D’Alessandro P., MacDonald P.B. (2024). The effect of tranexamic acid and epinephrine on visual clarity during arthroscopic shoulder surgery: a meta-analysis of RCTs. Orthop Traumatol Surg Res.

[50] Mayfield C.K., Liu K.C., Richardson M.K., Freshman R.D., Kotlier J.L., Fathi A. (2025). Tranexamic acid use in total shoulder arthroplasty continues to increase and is safe in high-risk patients. J Shoulder Elbow Surg.

[51] Myles P.S., Smith J.A., Forbes A., Silbert B., Jayarajah M., Painter T. (2017). Tranexamic acid in patients undergoing coronary-artery surgery. N Engl J Med.

[52] Narkbunnam R., Chompoonutprapa A., Ruangsomboon P., Udomkiat P., Chareancholvanich K., Pornrattanamaneewong C. (2021). Blood loss and transfusion rate compared among different dosing regimens of tranexamic acid administration in patients undergoing hip hemiarthroplasty for femoral neck fracture: a randomized controlled trial. Injury.

[53] Nugent M., May J.H., Parker J.D., Kieser D.C., Douglas M., Pereira R. (2019). Does tranexamic acid reduce knee swelling and improve early function following arthroscopic meniscectomy? A double-blind randomized controlled trial. Orthop J Sports Med.

[54] Okamoto S., Okamoto U. (1962). AMINO-METHYL-CYCLOHEXANE-CARBOXYLIC ACID: AMCHA. Keio J Med.

[55] Pearce K., Gibb J., Shetty S. (2024). VTE in trauma and orthopaedics. Surg Oxf.

[56] Poff C., Buckman V., Jung D., Liao C., Shi L., Maassen N.H. (2025). Administration of perioperative tranexamic acid and range of motion after total shoulder arthroplasty. J Shoulder Elb Arthroplasty.

[57] Qian Y., Yan J., Zhang S., Zhu S. (2025). Efficacy of tranexamic acid in improving visual clarity and operative time of arthroscopic rotator cuff repair: a systematic review and meta-analysis. Acta Orthop Traumatol Turc.

[58] Rana T., Mushtaq H.S., Memon K., Chan S., Kalogrianitis S. (2025). A retrospective study on the role of tranexamic acid in reverse total shoulder arthroplasty for trauma patients with complex proximal humerus fractures. Cureus.

[59] Richards J.E., Samet R.E., Koerner A.K., Grissom T.E. (2019). Tranexamic acid in the perioperative period. Adv Anesth.

[60] Richardson M.K., Liu K.C., Mayfield C.K., Kistler N.M., Lieberman J.R., Heckmann N.D. (2024). Tranexamic acid is safe in patients with a history of venous thromboembolism undergoing total joint arthroplasty. J Bone Joint Surg Am.

[61] Samborski S.A., Morris S.C., Leary S., Geiger K., Hlas A., Westermann R. (2025). Single-Dose intravenous tranexamic acid does not increase venous thromboembolic rate or complication rate during hip arthroscopy. Arthrosc J Arthrosc Relat Surg.

[62] Shi H., Ou Y., Jiang D., Quan Z., Zhao Z., Zhu Y. (2017). Tranexamic acid reduces perioperative blood loss of posterior lumbar surgery for stenosis or spondylolisthesis: a randomized trial. Medicine (Baltimore).

[63] Shu H.T., Mikula J.D., Yu A.T., Shafiq B. (2021). Tranexamic acid use in pelvic and/or acetabular fracture surgery: a systematic review and meta-analysis. J Orthop.

[64] Sun Y., Xiao D., Fu W., Cai W., Huang X., Li Q. (2022). Efficacy and safety of tranexamic acid in shoulder arthroscopic surgery: a systematic review and meta-analysis. J Clin Med.

[65] Suter T., McRae S., Zhang Y., MacDonald P.B., Woodmass J.M., Mutter T.C. (2024). The effect of intravenous tranexamic acid on visual clarity in arthroscopic shoulder surgery compared to epinephrine and a placebo: a double-blinded, randomized controlled trial. J Shoulder Elbow Surg.

[66] Takahashi R., Kajita Y., Iwahori Y., Harada Y. (2023). Tranexamic acid has no effect on postoperative pain control after arthroscopic rotator cuff repair: a prospective, double-blind, randomized controlled trial. Asia-pac J Sports Med Arthrosc Rehabil Technol.

[67] Tengborn L., Blombäck M., Berntorp E. (2015). Tranexamic acid – an old drug still going strong and making a revival. Thromb Res.

[68] Tripathy S.K., Varghese P., Kumarasamy A.K.N., Mishra N.P., Neradi D., Jain M. (2023). Safety and efficacy of tranexamic acid in hip hemiarthroplasty for fracture neck femur: a systematic review and meta-analysis. Indian J Orthop.

[69] Wang N., Xiong X., Xu L., Ji M., Yang T., Tang J. (2019). Transfusions and cost-benefit of oral versus intravenous tranexamic acid in primary total hip arthroplasty: a meta-analysis of randomized controlled trials. Medicine (Baltimore).

[70] Wang T.C., Guo J.L., Tian Q.P., Deng H.P., Yin B., Xiao Z. (2023). Application of tranexamic acid in shoulder arthroscopic surgery: a randomised controlled trial. Chin Med Sci J.

[71] Xiao C., Gao Z., Yu W., Yao K., Cao Y., Long N. (2023). Medullary cavity application of tranexamic acid to reduce blood loss in tibial intramedullary nailing procedures—a randomized controlled trial. Int Orthop.

[72] Xie B., Tian J., Zhou D. (2015). Administration of tranexamic acid reduces postoperative blood loss in calcaneal fractures: a randomized controlled trial. J Foot Ankle Surg.

[73] Yamaguchi Y., Urita A., Matsui Y., Tomizawa K., Iwasaki N., Taneichi H. (2025). Effect of topical tranexamic acid administration on postoperative blood loss and hematologic parameters after reverse shoulder arthroplasty. J Orthop Surg.

[74] Yang Y., Qin H., Zheng X., Hu B., Zhang M., Ma T. (2020). Administration of tranexamic acid in proximal humeral fractures. Indian J Orthop.

[75] Yoon J.P., Park S.J., Kim D.H., Lee H.J., Park E.J.J., Shim B.J. (2024). Tranexamic acid can reduce early Tendon adhesions after Rotator Cuff Repair and is not detrimental to Tendon-Bone healing: a comparative animal model study. Arthrosc J Arthrosc Relat Surg.

[76] Yoon J.Y., Park J.H., Kim Y.S., Shin S.J., Yoo J.C., Oh J.H. (2020). Effect of tranexamic acid on blood loss after reverse total shoulder arthroplasty according to the administration method: a prospective, multicenter, randomized, controlled study. J Shoulder Elbow Surg.

[77] Zhao J., Liang G., Huang H., Hong K., Pan J., Yang W. (2024). Intravenous tranexamic acid significantly improved visualization and shortened the operation time in Arthroscopic Rotator Cuff Repair: a systematic review and meta-analysis of level I and II studies. Arthrosc J Arthrosc Relat Surg.

[78] Zhou M., Li S., Zhang H., Lu Y. (2024). Does tranexamic acid reduce elbow swelling and improve early function following arthroscopic arthrolysis? A double-blind randomized controlled trial. J Shoulder Elbow Surg.

[79] Zhu R., Jiang H., Xu W., Shen L., Jin G. (2023). Impact of intra-articular injection with tranexamic acid on total blood loss and postoperative pain after arthroscopic rotator cuff repair surgery. Front Surg.

